# Worry, Rumination, and Metacognitive Beliefs in Adolescents with Obesity Associated with Binge Eating Disorder (BED) vs. Age-Matched Adolescents with Essential Obesity: A Cross-Sectional Study

**DOI:** 10.3390/jcm15020573

**Published:** 2026-01-10

**Authors:** Anna Guerrini Usubini, Maria Gobetti, Sara Ducale, Adele Bondesan, Diana Caroli, Francesca Frigerio, Laura Abbruzzese, Nicoletta Marazzi, Gianluca Castelnuovo, Alessandro Sartorio

**Affiliations:** 1 Experimental Laboratory for Auxo-Endocrinological Research, Istituto Auxologico Italiano, Istituto di Ricovero e Cura a Carattere Scientifico (IRCCS), 28824 Piancavallo-Verbania, Italy; a.bondesan@auxologico.it (A.B.); d.caroli@auxologico.it (D.C.); f.frigerio@auxologico.it (F.F.); n.marazzi@auxologico.it (N.M.); sartorio@auxologico.it (A.S.); 2 Psychology Research Laboratory, Istituto Auxologico Italiano, Istituto di Ricovero e Cura a Carattere Scientifico (IRCCS), 28824 Piancavallo-Verbania, Italy; m.gobetti@auxologico.it (M.G.); s.ducale@auxologico.it (S.D.); gianluca.castelnuovo@auxologico.it (G.C.); 3 Division of Auxology, Istituto Auxologico Italiano, Istituto di Ricovero e Cura a Carattere Scientifico (IRCCS), 28824 Piancavallo-Verbania, Italy; l.abbruzzese@auxologico.it; 4 Department of Psychology, Catholic University of Milan, 20123 Milan, Italy

**Keywords:** worry, rumination, metacognitive beliefs, metacognition, adolescence, obesity, binge eating disorder

## Abstract

**Background/Objectives:** The study aimed to investigate the presence of worry, rumination, and metacognitive beliefs in adolescents with maladaptive eating behaviours. **Methods:** The study involved 37 adolescents (10 males, 27 females, mean age ± SD: 15.4 ± 1.53 years) with obesity (Body Mass Index, BMI > 97th centile) associated with binge eating disorder (BED) (BES score ≥ 17) and 30 age-matched adolescents (13 males, 17 females, mean age ± SD: 15.2 ± 1.98 years) with essential obesity (i.e., without BED, BES score < 17). Participants completed self-report questionnaires—Penn State Worry Questionnaire (PSWQ), Ruminative Response Scale (RRS), Anger Rumination Scale (ARS), and Metacognitions Questionnaire for Children (MCQ-C)—to assess binge eating, worry, rumination, and metacognitive beliefs, respectively. **Results:** Patients with obesity and BED showed higher scores on the PSWQ (*p* = 0.006), RRS (*p* < 0.001), ARS (*p* < 0.001), negative Metaworry (*p* = 0.011), and total MCQ-C (*p* = 0.027) than those with essential obesity, with a medium-to-large effect size, indicating that the differences between subgroups were meaningful. **Conclusions:** Our findings highlight that BMI alone is not associated with metacognitive processes and beliefs. The presence of BED in adolescents with obesity is linked to increased levels of worry, rumination, and maladaptive metacognitive beliefs, in comparison with age-matched adolescents with essential obesity. The results of the study underline the need for different psychological approaches in these clinical conditions going forward.

## 1. Introduction

Adolescence is a critical developmental stage characterized by significant physical, emotional, and cognitive changes. During this period, mental health vulnerabilities often emerge, with obesity and binge eating disorder (BED) being among the most prevalent concerns. According to the World Health Organization [1], in 2022, 43% of adults aged 18 years and over were overweight, and 16% were obese. Overweight and obesity represent a relevant medical and social problem, including in childhood, with over 390 million children and adolescents aged 5–19 years being overweight and obese [2,3].

Obesity, defined as an excessive accumulation of body fat, is a multifaceted condition associated with severe physical and psychological complications, including depression, anxiety, and poor self-esteem. Being overweight and obese is often associated with binge eating disorder (BED) [4]. A binge eating episode consists of eating a greater amount of food than most people would in a short time, with a perceived loss of control [5]. Feelings of guilt, shame, and distress characterize these symptoms, due to the perceived uncontrollability of eating and its consequences [6]. Binge eating episodes are common in different eating disorders [7], often associated with psychological distress, mental health conditions, and other clinical conditions [8].

Obesity during adolescence is associated with several health consequences, which include prediabetes and type 2 diabetes, non-alcoholic fatty liver disease [9], dyslipidemia [10], polycystic ovary syndrome [11], obstructive sleep apnea [12], mental health disorders, and social stigma [13]. In addition, obesity during adolescence is a risk factor for complications in adulthood [14,15]. The risk of the onset of binge eating increases during late adolescence [12], with girls showing a higher prevalence compared to boys. Binge eating episodes during adolescence are also a risk factor for the subsequent onset of other eating disorders during adulthood [13].

Overeating episodes are not merely excessive food intake but are accompanied by a loss of control and psychological distress. Several hypotheses have been advanced to explain the onset and development of overeating in adolescents. Maladaptive eating behaviours often occur in response to negative emotional states, leading to a vicious cycle of overeating, guilt, and further emotional distress. According to the affect regulation model, individuals engage in behaviours such as overeating to temporarily reduce emotional distress, even though these actions may lead to long-term negative consequences [16].

In addition, cognitive processes have been identified to be central in determining maladaptive eating [17]. Worry and rumination are defined as cognitive processes characterized by a repetitive, frequent, and self-focused form of thinking [18]. Worry is described as a series of thoughts and mental images associated with negative feelings and experienced as relatively uncontrollable. It represents an effort to mentally solve problems related to uncertain outcomes, often with a potentially negative result. In contrast, rumination involves persistent thoughts that centre on negative emotions, symptoms, and their causes, meanings, and consequences. Rumination can occur in both verbal and visual forms, often characterized by repetitive focus on oneself, distressing events, and personal issues. While worry tends to be more future-oriented and problem-solving in nature, rumination frequently revolves around themes of loss and is focused on difficulties in the past [19]. These processes can be systematically explored using validated self-report measures that capture both their content and underlying regulatory mechanisms. Specifically, worry can be assessed with the Penn State Worry Questionnaire (PSWQ), which measures the tendency to engage in excessive and uncontrollable worry, whereas rumination can be examined using the Anger Rumination Scale (ARS) and the Ruminative Response Scale (RRS), which assess repetitive thinking focused on anger-related experiences and depressive mood, respectively. An extensive literature base suggests that both worry and rumination are cognitive processes present across diverse disorders, including overeating [20,21]. The metacognitive model identified five dysfunctional processes based on negative and positive evaluations of worry and rumination, on attempts to monitor and suppress thoughts, and on low confidence in own cognitive functions [22]. These metacognition processes lead to maladaptive cognitive responses and ineffective coping strategies, contributing to psychological distress [23]. These beliefs are particularly relevant in adolescents, whose cognitive and emotional regulatory systems are still maturing, making them more susceptible to maladaptive thought patterns. Metacognitive beliefs—the beliefs individuals hold about their own thinking processes—can be evaluated using the Metacognitions Questionnaire (MCQ), allowing investigation of how such beliefs may contribute to the maintenance of worry and rumination.

Despite the growing recognition of the role of cognitive and metacognitive processes in maladaptive eating behaviours, the relationship between worry, rumination, metacognitive beliefs, and overeating in adolescents remains underexplored. The primary research question of this study was whether adolescents with obesity and comorbid binge eating disorder (BED) differ from adolescents with essential obesity in levels of worry, rumination, and metacognitive beliefs. Accordingly, the objectives of this study were (i) to assess levels of worry, rumination, and metacognitive beliefs in adolescents with maladaptive eating behaviours and (ii) to compare these cognitive processes between adolescents with obesity and BED and those with essential obesity. Based on previous evidence linking worry and rumination to the development and maintenance of BED [24,25,26], we hypothesized that adolescents with obesity and BED would exhibit higher levels of worry, rumination, and dysfunctional metacognitive beliefs than their counterparts with essential obesity.

## 2. Materials and Methods

### 2.1. Participants and Procedures

Participants were 37 adolescents with obesity and BED, diagnosed according to the score of the Binge Eating Scale (BES score ≥ 17) and confirmed with a semi-structured clinical interview according to the DSM-5 criteria conducted by a clinical psychologist (10 males, 27 females, mean age ± SD: 15.4 ± 1.53 years, Body Mass Index: BMI > 97th centile) and 30 age-matched individuals with essential obesity (13 males and 17 females, mean age ± SD: 15.2 ± 1.98 years; BES score < 17). Participants were recruited at the Division of Auxology, Istituto Auxologico Italiano IRCCS, Piancavallo (VB), a third-level medical and research centre for in-hospital multidisciplinary metabolic rehabilitation of severe obesity. Inclusion criteria were (1) Italian nationality, (2) age between 11 and 17 years, and (3) a Body Mass Index above the 97th percentile for sex and chronological age, based on the Italian growth charts [27]. Individuals with endocrine obesity (i.e., hypothyroidism, Cushing syndrome) or syndromic obesity (Prader–Willi, Bardet–Biedl, Down) or individuals with any form of physical or mental impairment that could compromise the compilation of the questionnaires were excluded from the study population.

After receiving information about the study and after written informed assent was provided by the adolescents and written consent by their parents, participants were screened through a clinical interview. Following enrollment, eligible participants were asked to provide sociodemographic information and complete self-report questionnaires. Recruitment took place between January and July 2024.

The current study was approved by the Ethical Committee of Istituto Auxologico Italiano, IRCCS, Milan, Italy (research project code: 03C121; acronym: ACTyouCHANGE-Adolescents). Research was carried out according to the Declaration of Helsinki and its advancements.

### 2.2. Measures

Socio-demographic data: Participants were asked to provide information about their gender, age, and educational level. Such information was self-reported.

Anthropometric data: The internal medical staff measured weight and height to calculate Body Mass Index (BMI) according to the proper formula: kg/m^2^. Standing height was determined by a Harpenden Stadiometer (Holtain Limited, Crymych, Dyfed, UK). Weight was measured to the nearest 0.1 kg using an electronic scale (RoWU 150, Wunder Sa.bi., Trezzo sull’Adda, Italy). Waist circumference (WC) was measured in a standing position with a non-elastic flexible tape measure, with participants gently exhaling midway between the lowest rib and the top of the iliac crest [28]. Fat-free Mass (FFM) was calculated using the prediction equation [29], and Fat Mass (FM) was derived as the difference between Body Mass and FFM.

Binge eating: The Binge Eating Scale was used to assess the presence and severity of binge eating behaviours, thus allowing the division of the study population into two subgroups (adolescents with obesity associated with BED and adolescents with essential obesity). The BES is a self-report questionnaire developed by Gormally et al. [30]; it consists of 16 items that evaluate behavioural, emotional, and cognitive aspects associated with binge eating episodes. Each item includes a set of statements ranked by severity, and respondents are instructed to select the statement that best describes their experiences. Scores range from 0 to 46, with higher scores indicating more severe binge eating tendencies. Scores higher than 27 indicate severe binge eating tendencies, strongly indicative of a potential diagnosis of binge eating disorder (BED). The Italian version [31] has demonstrated good psychometric properties in terms of validity and reliability in previous studies [32]. In our sample, the scale’s internal consistency was excellent (α = 0.93).

Worry: The Penn State Worry Questionnaire (PSWQ; [33]) is a widely used self-report instrument designed to assess the tendency and intensity of worry, which is a central feature of generalized anxiety disorder. The Italian version of the PSWQ [34] maintains the original 16-item structure, with responses rated on a 5-point Likert scale ranging from 1 (not at all typical of me) to 5 (very typical of me). Higher total scores indicate a greater tendency to engage in excessive and uncontrollable worry. The Italian version has demonstrated strong psychometric properties, including high internal consistency and satisfactory construct validity in various populations [35,36]. The scale’s internal consistency in our sample was excellent (α = 0.93).

Rumination: The Ruminative Response Scale (RRS; [37]) is a widely used self-report measure of the tendency to ruminate in response to depressed mood. The RRS comprises 22 items, which are rated using a 4-point Likert scale (1 = “Almost never” and 4 = “Almost always”). Higher scores indicate higher levels of rumination. The RRS has demonstrated good reliability and validity across clinical and community samples [38]. The scale’s internal consistency in our sample was excellent (α = 0.94).

The Anger Rumination Scale (ARS; [39]) is a self-report measure developed to assess the tendency to engage in rumination focused on anger. The Italian version by Baldetti & Bartolozzi [40] consists of 14 items rated on a 4-point Likert scale (from 1 = almost never to 4 = almost always); in the Italian sample, the scale showed good internal consistency (α = 0.85) and test–retest reliability (r = 0.84). The scale’s internal consistency in our sample was good (α = 0.88).

Metacognitive beliefs: The Metacognitions Questionnaire for Children (MCQ-C; [41]) is a widely used self-report measure of metacognitions in children and adolescents. It comprises 24 items, which are rated using a 4-point Likert scale (1 = “Do not agree” and 4 = “Agree very much”), addressing 4 subscales: positive beliefs about worry (Positive Metaworry); negative beliefs about worry (Negative Metaworry); superstitious, punishment, and responsibility beliefs (SPR beliefs); and awareness of one’s thoughts (Cognitive Monitoring). The MCQ-C demonstrated good internal consistency reliability, as well as concurrent and criterion validity, and four valid factors. The Italian version was adapted by Benedetto and Di Blasi [42]. Scores range from 24 to 96 points, and higher scores indicate greater negative metacognitive activity. The internal consistency (Cronbach’s alphas) of the Italian version of the MCQ-C, ranged from 0.61 (Positive Metaworry scale) to 0.78 (Negative Meta-worry scale). In our sample, the internal consistency of the scales (α) ranged from 0.63 (Positive Metaworry) to 0.84 (Negative Metaworry).

### 2.3. Statistical Analysis

An a priori power analysis was conducted using G*Power (version 3.1) to determine the required sample size for a one-tailed independent-samples *t* test. Assuming a significance level of α = 0.05, a medium effect size (Cohen’s *d* = 0.70), and a statistical power of 0.80, the analysis indicated that a total sample size of 52 participants was sufficient to correctly reject the null hypothesis.

Descriptive statistics of the study variables were calculated properly. Skewness and Kurtosis indices were applied to verify whether the variables had a normal distribution. Correlations between the study variables were performed using Pearson’s r coefficient. To compare data of patients with obesity and BED vs. those with obesity without BED on the PSWQ, RRS, ARS, and MCQ, independent-sample *t*-tests were performed. Critical alpha was set at 0.05. Cohen’s *d* was used as a measure of effect size, with the following interpretation: *d* = 0.2: small effect; *d* = 0.5: medium effect; *d* = 0.8: large effect [43].

Analyses have been performed using Jamovi (2.6.26).

## 3. Results

The total sample of the study comprised 67 consecutive Italian adolescents with obesity (23 males, 44 females), mean age ± SD: 15.3 ± 1.74 years; mean Body Mass Index (BMI): 39.6 ± 7.53: kg/m^2^; mean WC ± SD: 112.6 ± 17.76; mean FFM ± SD: 57.6 ± 11.67 Kg; mean FM ± SD: 50.5 ± 20.27. Most of them lived in Northern Italy (89%) and attended secondary school (67%).

The study population was evaluated with the BES to obtain two subgroups, one with obesity associated with BES (BES score ≥ 17) and the other with essential obesity without BES (BES score < 17).

Thirty-seven adolescents were diagnosed with BED (mean ± SD BES score: 27.22 + 7.45). In this group, 10 (27%) were males, and 27 were females (73%), mean age ± SD: 15.4 ± 1.53 years; mean Body Mass Index (BMI): 40 ± 6.65: kg/m^2^; mean WC ± SD: 115.9 ± 11.22 cm; mean FFM ± SD: 59.03 ± 8.85 kg; mean FFM ± SD: 53.61 ± 4.99%; mean FM ± SD: 51.58 ± 11.11 kg; mean FM ± SD: 46.39 ± 4.99%.

Thirty adolescents were diagnosed with essential obesity (without BED, mean ± SD BES score: 7.97 ± 5.16): 13 males and 17 females, mean age ± SD: 15.2 ± 1.98 years, mean Body Mass Index (BMI): 39.2 ± 8.55: kg/m^2^; mean WC ± SD: 112.63 ± 17.76 cm; mean FFM ± SD: 57.58 ± 11.66 kg; mean FFM ± SD: 54.37 ± 6.27%; mean FM ± SD: 50.55 ± 20.26 kg; mean FM ± SD: 45.64 ± 6.28%.

The two subgroups did not show differences in mean weight (*p* = 0.68) and mean BMI (*p* = 0.65), WC (*p* = 0.36) FFM (kg) (*p* = 0.58) FFM (%) (*p* = 0.60), FM (kg) (*p* = 0.80), or FM (%) (*p* = 0.60).

Descriptive statistics of the sample are reported in Table 1.

Pearson’s correlations were performed to assess the associations between the study variables. The results showed no significant associations between BMI and all the other study variables, including BES, PSWQ, RRS, ARS, and MCQ-C (subscales and total score). In contrast, BES showed positive and significant associations with all the study variables, except for the Positive Metaworry subscale of MCQ-C (*p* = 0.146). Correlations are presented in Table 2.

Group comparisons revealed significant differences between adolescents with obesity and BED and those with essential obesity (without BED) in terms of cognitive processes. Specifically, participants with obesity and BED showed significantly higher levels of worry, rumination, and metacognitive beliefs compared to their counterparts with essential obesity. In particular, patients with obesity and BED showed higher scores on the PSWQ (*p* = 0.006), RRS (*p* < 0.001), ARS (*p* < 0.001), negative Metaworry (*p* = 0.011), and total MCQ-C (*p* = 0.027) than those with essential obesity, with a medium to large effect size, suggesting that differences between groups were meaningful. Comparisons are reported in Table 1.

## 4. Discussion

The present study aimed to investigate the presence of worry, rumination, and metacognitive beliefs in adolescents with obesity associated with binge eating disorder (BED) and in age-matched adolescents with essential obesity.

Our findings primarily indicate that BMI does not correlate with metacognitive processes of worry and rumination and with the assessed metacognitive beliefs. This result contrasts with the current literature, which suggests a possible association between these factors. For example, a study conducted on 155 high school students in Isfahan, Iran, examined the correlation between body dysmorphic disorder and metacognitive subscales, including metaworry and cognitive fusion. The results indicated a significant correlation between body dysmorphic disorder scores and metacognitive measures, suggesting a relationship between dysfunctional metacognitive beliefs and concerns about body image in adolescents [44]. Additionally, a meta-analysis examined the role of worry and rumination in eating disorders, highlighting a significant relationship between repetitive negative thinking and eating disorders. Although this analysis did not focus exclusively on adolescents, it suggested a possible association between these metacognitive processes and eating-related issues [19]. Furthermore, a study conducted by Spada et al. [45] examined how negative metacognitions about craving predicted the severity of binge eating in women. The results indicated that such metacognitions were significantly correlated with self-reported BMI, negative mood, irrational eating beliefs, and craving, suggesting a relationship between dysfunctional metacognitive beliefs and disordered eating behaviours. However, this study did not focus on adolescents, and in general, it is essential to note that research specifically addressing the association between BMI and metacognitive processes in adolescents is limited. Therefore, further studies are needed to clarify the nature of these relationships in this group. The lack of a significant correlation between BMI and metacognitive processes observed in our study may also be attributed to the limited variability in BMI within our sample, totally composed of patients with obesity. A restricted range of BMI values can reduce statistical power, potentially masking existing associations between the study variables [46].

As far as comparisons between adolescents with obesity and BED and those with essential obesity are concerned, our results showed significant differences in metacognitive processes. Specifically, adolescents with obesity and comorbid BED exhibited higher levels of worry and rumination compared to those without BED. This result is consistent with previous findings suggesting an association between worry, rumination, and eating disorders [19,45,47,48,49,50]. Additionally, these patients demonstrated stronger negative metacognitive beliefs related to the uncontrollability of worry (negative Metaworry) than those with essential obesity. These results align with and extend to adolescents with BED the existing literature, which found that individuals with anorexia nervosa and bulimia nervosa showed higher levels of metacognitive beliefs about the danger and uncontrollability of thoughts, the need to control thoughts, and cognitive self-consciousness compared to healthy controls [51,52,53].

Focusing on an understudied population, our findings highlight that while BMI alone does not appear to be associated with metacognitive processes and beliefs, the presence of BED in adolescents with obesity is linked to higher levels of worry, rumination, and maladaptive metacognitive beliefs.

This study has several limitations. First, the cross-sectional design does not allow causal inferences among the variables. Second, the use of self-report measures may have introduced individual response biases. Additionally, the questionnaires were originally developed for adult populations. Moreover, most participants were recruited from a single clinical centre in Northern Italy, limiting the generalizability of the findings and highlighting the need for replication in more diverse cultural and geographical contexts. Finally, the imbalance between male and female participants represents a further limitation, as it precluded the examination of potential gender differences in the study variables.

Despite these limitations, this is one of the few studies about metacognitive processes and beliefs and eating disorders in adolescents with obesity and BED. Future research should explore the causal relationships between metacognition and overeating to elucidate further the mechanisms linking these cognitive processes with disordered eating behaviours. A more comprehensive understanding of the role of metacognitive variables in eating behaviours may have important clinical implications, as it could inform the development of more targeted and effective interventions for eating disorders. Specifically, identifying maladaptive metacognitive beliefs and dysfunctional cognitive processes that contribute to the onset and maintenance of binge eating behaviours would allow clinicians to directly reduce the psychological mechanisms of binge eating disorder (BED).

In this context, therapeutic approaches aimed at modifying maladaptive metacognitive beliefs and reducing dysfunctional cognitive processes—such as Metacognitive Therapy (MCT) [54]—may be particularly promising. MCT focuses on decreasing cognitive perseveration, including rumination and worry, and on fostering more adaptive coping strategies and greater cognitive flexibility.

In conclusion, this study highlights that metacognitive processes, rather than BMI alone, are crucial in distinguishing adolescents with obesity and BED from those with essential obesity. Adolescents with obesity and BED showed higher levels of worry, rumination, and maladaptive metacognitive beliefs. Clinically, these findings support the inclusion of metacognitive assessment and interventions—such as adapted Metacognitive Therapy—within multidisciplinary treatment programmes to target the cognitive processes maintaining binge eating behaviours. Future research should employ longitudinal and intervention designs, use adolescent-specific measures, and include more diverse and balanced samples to clarify causal relationships, gender effects, and cultural influences on metacognition and binge eating.

## Figures and Tables

**Table 1:** Descriptive statistics and comparisons between adolescents with essential obesity and patients with obesity and BED.

	Adolescents with Essential Obesity (*n* = 30)	Adolescents with Obesity and BED (*n* = 37)	Group Comparison	
	M (SD)	M (SD)	t (*p*)	Cohen’s d Effect Size
PSWQ	45.13 (11.77)	54.51 (14.46)	2.86 (0.006)	0.704
RRS	43.73 (12.42)	57.68 (14.53)	4.16 (<0.001) *	1.023
ARS	28.73 (7.65)	35.70 (7.68)	3.70 (<0.001) *	0.909
MCQ-C tot	47.43 (9.83)	52.97 (10.06)	2.26 (0.027) *	0.556
MCQ-C Positive Metaworry	8.90 (2.32)	9.81 (2.87)	1.44 (0.155)	0.353
MCQ-C Negative Metaworry	8.33 (3.25)	10.54 (3.53)	2.63 (0.011) *	0.647
MCQ-C SPR Beliefs	13.30 (4.19)	14.57 (4.53)	1.18 (0.243)	0.289
MCQ-C Cognitive monitoring	16.90 (4.03)	18.05 (3.88)	1.19 (0.238)	0.292

Note: PSWQ: Penn State Worry Questionnaire; RRS: Ruminative Response Scale; ARS: Anger Rumination Scale; MCQ-C: Metacognitions Questionnaire for Children; SPR: superstitious, punishment, and responsibility; * *p* < 0.05.

**Table 2:** Correlations between the study variables.

	BMI	BES	PSWQ	RRS	ARS	MCQ-C Tot	MCQ-C Positive Metaworry	MCQ-C Negative Metaworry	MCQ-C SPR Beliefs	MCQ-C Cognitive Monitoring
BMI	-									
BES	0.174 (*p* = 0.163)	-								
PSWQ	−0.101 (*p* = 0.418)	0.435 * (*p* < 0.001)	-							
RRS	−0.001 (*p* = 0.994)	0.621 * (*p* < 0.001)	0.774 * (*p* < 0.001)	-						
ARS	−0.025 (*p* = 0.844)	0.432 * (*p* < 0.001)	0.512 * (*p* < 0.001)	0.568 * (*p* < 0.001)	-					
MCQ-C tot	0.027 (*p* = 0.829)	0.439 * (*p* < 0.001)	0.661 * (*p* < 0.001)	0.712 * (*p* < 0.001)	0.488 * (*p* < 0.001)	-				
MCQ-C Positive Metaworry	−0.020 (*p* = 0.873)	0.180 (*p* = 0.146)	0.182 (*p* = 0.141)	0.106 (*p =* 0.394)	0.027 (*p* = 0.830)	0.360 * (*p* = 0.003)	-			
MCQ-C Negative Metaworry	−0.033 (*p* = 0.790)	0.465 * (*p* < 0.001)	0.775 * (*p* < 0.001)	0.690 * (*p* < 0.001)	0.533 * (*p* < 0.001)	0.816 * (*p* < 0.001)	0.139 (*p* = 0.261)	-		
MCQ-C SPR Beliefs	0.006 (*p* = 0.960)	0.304 * (*p* = 0.012)	0.456 * (*p* < 0.001)	0.581 * (*p* < 0.001)	0.416 * (*p* < 0.001)	0.805 * (*p* < 0.001)	0.003 (*p* = 0.983)	0.653 * (*p* < 0.001)	-	
MCQ-C Cognitive monitoring	0.108 (*p* = 0.386)	0.265 * (*p* = 0.030)	0.391 * (*p* < 0.001)	0.511 * (*p* < 0.001)	0.308 * (*p* < 0.001)	0.729 * (*p* < 0.001)	0.149 * (*p* = 0.228)	0.389 * (*p* = 0.001)	0.389 * (*p* < 0.001)	-

Note: BES: Binge Eating Scale; PSWQ: Penn State Worry Questionnaire; RRS: Ruminative Response Scale; ARS: Anger Rumination Scale; MCQ-C: Metacognitions Questionnaire for Children; SPR: superstitious, punishment, and responsibility; * *p* < 0.05.

## Data Availability

Raw data will be uploaded on www.zenodo.org immediately after the manuscript is accepted and they will be available upon reasonable request from authors A.G.U. and A.S.
